# Cumulative Incidence of Mental Disorders Among German Military Personnel Deployed to Iraq 2015–2018—An Epidemiological Study

**DOI:** 10.3390/ejihpe15050081

**Published:** 2025-05-14

**Authors:** Ulrich Wesemann, Karl-Heinz Renner, Harald Hofmann, Nils Hüttermann, Gerd-Dieter Willmund

**Affiliations:** 1 Department of Psychiatry, Psychotherapy and Psychotraumatology, Bundeswehr Hospital Berlin, Scharnhorststr. 13, 10115 Berlin, Germany; 2 Institute of Psychology, Faculty of Human Sciences, Bundeswehr University Munich, 85577 Neubiberg, Germany; 3 Bundeswehr Operations Command, Henning-von-Tresckow-Kaserne, 14548 Schwielowsee, Germany

**Keywords:** Iraq, mental health, PTSD, latency, military personnel, cumulative incidence, epidemiology

## Abstract

**Background:** There is currently no study examining the mental health consequences of deployed German service members in Iraq. The aim is, therefore, to determine the cumulative incidence and latency period until the first diagnosis of deployment-related mental disorders. We hypothesized a lower rate than for Afghanistan with 2.4%. **Methods:** All registered *N* = 1635 German military personnel who were deployed to the mission “Training support contingent Northern Iraq” between 2015 and 2018 were included. Individuals with mental disorders attributed to this deployment were identified in a central database. Differences in latency between diagnoses were calculated by *t*-tests for independent samples. **Results:** By January 2024, *n* = 55 (3.4%) individuals had been recorded who developed a mental disorder as a result of this deployment. Most of them (54.5%) had a post-traumatic stress disorder (PTSD) as the main or comorbid diagnosis. There were no gender differences in the cumulative incidence (male 3.6%; female 2.9). The latency period between the onset of disease and initial diagnosis was, on average, 1.0 years (standard deviation 1.1 years; Q_25_ < 1 year, Q_50_ = 1 year and Q_75_ = 2 years). With 1.3 vs. 0.6 years, the latency was significantly longer for individuals with PTSD. **Conclusions:** The cumulative incidence appears to be higher after the Iraq deployment than after most other Bundeswehr deployments. This is attributed to particular threats. The latency period is higher for those affected with PTSD than with other disorders. This could be due to a higher degree of stigmatization. It is, therefore, worthwhile to compare the different deployments in order to be able to derive better preventive and aftercare measures as well as destigmatization programs to prevent chronification.

## 1. Introduction

Military deployments bear a high risk of developing physical (Alruwaili et al., 2023; Khorram-Manesh et al., 2022; Teplova et al., 2022) and mental disorders (Boyle et al., 2024; Inoue et al., 2024; Knaust et al., 2023). Therefore, mental disorders can be considered an occupational hazard for military and other emergency service personnel (Sahebi et al., 2021; Sahebi et al., 2020; Tahernejad et al., 2023). While there has been a focus on post-traumatic stress disorders (PTSD) in the past three decades, recent research is taking a wider range of mental disorders, like anxiety disorders, depression, or substance use disorders, into account (Finnegan & Randles, 2023; Iversen et al., 2009; Muysewinkel et al., 2024). Additionally, mental health symptoms like problematic anger (MacManus et al., 2015; Nordstrand et al., 2024a; Sinai et al., 2018), grief (Xu et al., 2022), suicidality (Willmund et al., 2019a, 2019b), or sleep problems (Danker-Hopfe et al., 2018) are seen as early behavioral indicators for the later onset of mental disorders (Wesemann et al., 2017). The same is true for changes in cytokines such as TNF-a (Himmerich et al., 2015, 2016). Circumstances like proximity to the scene (Motreff et al., 2020) or in time (Wesemann et al., 2021) are seen as proxies for a perceived threat. Similarly, different occupational groups are the proxies of different tasks on-site, leading to different emotions in major disasters (Wesemann et al., 2020). Other important research topics include education and training (Kasselmann et al., 2020), intervention (Brand et al., 2011), gender (Dell et al., 2024), social support (Grover et al., 2024), family stress (Senior et al., 2023), personality factors (Prykhodko, 2022), or resilience (Niederhauser et al., 2023; Sefidan et al., 2021; Ziehr & Merkt, 2024; Zueger et al., 2023). In summary, the field of research has become broader and many different circumstances can contribute to the development of mental symptoms or disorders.

The German Armed Forces (Bundeswehr) training support for the Peshmerga in Iraq represents Germany’s contribution to the international stabilization of the Middle East. Since 2014, the Federal Republic of Germany has been involved in the international anti-IS coalition to support the Kurdish armed forces, known as the Peshmerga, in the fight against the so-called Islamic State (IS). This training mission aimed to improve the military capabilities of the Peshmerga, strengthen their resilience, and thus contribute to long-term security and stability in the region. German support included not only the teaching of tactical and technical skills, but also the supply of equipment and humanitarian aid. This included training in areas such as logistics, medical care, and engineering. A particular focus was placed on strengthening the professionalism and resilience of the Iraqi armed forces. Military personnel also provided intelligence support and promoted cooperation between the various regional armed forces. They were primarily deployed in the semi-autonomous Kurdish region of Iraq, particularly around Erbil. The mission “Training Support Contingent Northern Iraq” formally ended with the merger with the second mandate of Operation Inherent Resolve (OIR) Counter DAESH in April 2018 to form Mission C-DAESH.

Although the military mission was clearly focused on combating terrorism, it also aimed to promote stability and peace. Soldiers, therefore, had to balance military objectives with the promotion of human rights and civil protection, which represented a cultural component of their missions. This deployment exposed them to unique and intense stressors, including proximity to the U.S. Embassy, which was frequently targeted, a tense security environment, and complex geopolitical circumstances. These conditions may contribute to an increased risk for developing mental health problems. In summary, the deployment reflects the complexity of contemporary military operations. It required a balance between operational effectiveness, geopolitical considerations, and cultural awareness.

By accurately assessing the cumulative incidence of mental health disorders, it is possible to better understand the extent of the impact on the military personnel’s mental health and thus develop a more informed approach to mental health care and support systems.

The cumulative incidence of mental disorders can help identify specific vulnerabilities in the Iraq deployment compared to other deployments. Different types of deployments, such as combat, peacekeeping, or humanitarian assistance, have different risk profiles. Service members who experience mission-specific life-threatening incidents are more likely to develop PTSD, depression, or anxiety disorders (Wesemann et al., 2024). The risk profiles can, therefore, be used to tailor interventions to the individual needs of different groups. This nuanced understanding is critical to improving the overall mental resilience of service members. It can help implement targeted pre- and post-deployment strategies that can reduce the risk of developing mental disorders.

Previous research investigating the mental health impact of an Afghanistan mission resulted in a one-year incidence rate for PTSD with 2.2–2.3% (Wittchen et al., 2012). For depression, it was 2.4%; for anxiety disorders, 5.1%; and for multimorbidity, defined as PTSD and depression or PTSD and an anxiety disorder, 1.3% (Wesemann et al., 2024). Compared to the 20 years in Afghanistan, this part of the four-year mission of the Training Support Contingent in northern Iraq was relatively small and short. The missions also differ in that, although the Iraq mission was “only” a training mission, the German base was frequently exposed to external threats such as rocket fire due to its proximity to a US embassy.

This makes the German deployment significantly different from that of the US. US military personnel were found to have significantly more combat experience in Iraq than in Afghanistan. This is also associated with higher rates of anxiety, depression, and PTSD in combat infantry units (Hoge et al., 2008). Another study found similar results regarding combat stress and mental health disorders among US veterans from Iraq and Afghanistan. Compared to all other deployments, combat stress was most severe in Iraq and Afghanistan, leading to the highest rates of anxiety, depression, and post-traumatic stress disorder (Na et al., 2023). Among UK military personnel, 35% reported relationship problems and 3.4 percent reported interpersonal violence after their deployments in Iraq and Afghanistan (Lane et al., 2022).

From this and the circumstances described above, no cumulative incidence of mental disorders as a result of the Iraq deployment can be derived a priori.

As there are still no data on the mental health impact of the German Iraq mission, the aim of this study is to determine the cumulative incidence rate of all military personnel with mental disorders attributed to this deployment. A secondary aim is to calculate the latency time between the onset of the disorder and the initial diagnosis and gender differences in this help-seeking behavior. The hypothesis is that the cumulative incidence rate in Iraq is lower than in Afghanistan. Furthermore, we hypothesize that the latency period for PTSD is higher than for all other mental disorders. Finally, we hypothesize that there is a longer latency period for female military personnel. This is assumed because under-represented groups often have to work harder to receive equal recognition (Kanter, 1977).

## 2. Methods

To calculate the cumulative incidence, it is necessary to examine how many military personnel were deployed and how many of them suffer from a deployment-related mental disorder. It is calculated as the percentage of military personnel with a mental disorder from that deployment. The cumulative incidence was defined as the percentage of affected personnel compared to the total deployed personnel. The time of initial diagnosis was set to at least five years after deployment (end of deployment 2018—end of inclusion 2023). This means that all individuals identified within 0 (directly diagnosed) to 9 years (onset of disease in 2014 and initial diagnosis in 2023) after deployment were included in the calculation of the cumulative incidence. Since this is a complete survey, there are no exclusions of cases.

### 2.1. Data Acquisition of the Full Survey (How Many Were Deployed?)

The Bundeswehr Operations Command (BwJFOCOM) is responsible for all logistic aspects concerning military deployments. Therefore, they have all information about individuals deployed. BwJFOCOM was asked to provide the number of German military personnel deployed between 2015 and 2018 as part of the Training Support Contingent Northern Iraq mission. Additionally, a breakdown into specific subgroups was asked for. In response to this inquiry, it was reported that *N* = 1635 military personnel were involved in this operation.

All *N* = 1635 German military personnel (*n* = 108 female; 6.6%) who were deployed in Iraq between 2015 and 2018 were included in this study. According to their rank, they were categorized in one of three groups: enlisted soldiers (NATO-Rangcode OR 1–4), sergeants (OR 4/5–9), and officers (OF1+). The demographic data including sex are given in Table 1. There is no information about gender orientation; nevertheless, diverse gender was not recorded. In the following, we will report about gender and not sex differences, as we expect a higher influence of the social role (Jones et al., 2019).

### 2.2. Measures—Procedure of the Diagnostic Process by the Specialists

The diagnosis of mental disorders in military personnel is made by psychiatrists according to the ICD-10 classification. This is part of routine psychiatric care in Bundeswehr hospitals or specialized centers. The reasons for referral are not recorded in the statistics described below; the initiative usually lies with the affected individuals themselves. In addition, the specialist asks about the onset of symptoms and documents this. Although the procedures in most Bundeswehr hospitals and specialist centers are similar, there is no standardized workflow across all areas. In almost all cases, however, different validated questionnaires are used in addition to the medical exploration. These questionnaires assess common deployment-related mental disorders such as anxiety, depression, and PTSD. Since these psychiatrists are specialists, the diagnosis can be considered the gold standard.

If a connection with a military deployment is suspected during the diagnostic process, further data will be collected. As part of the exploration, the patient’s deployment affiliation is also inquired about, and a specialist assesses whether the critical operational event is the cause of the disorder. In addition to the exploration, the “Troop in Contact” (TIC) form is also used. Military psychologists directly at the deployment site complete the TIC form. It contains a rough description of the critical operational event and confirms that the military member was present at the event. The entire process is a clinical, not a legal, assessment.

### 2.3. Data Collection on Military Personnel with Mental Disorders Due to the Iraq Deployment 2015–2018

The Center for Psychotraumatology at the Bundeswehr Hospital in Berlin hosts a central database called “deployment statistics”. In this database, all service members with deployment-related mental disorders are assessed. The data are reported by the psychiatrists at all Bundeswehr hospitals and all military specialist offices. An entry in the database is only made if the diagnosis is confirmed and has a clear connection to one of the deployments. It is, therefore, highly likely that the disorder would not have arisen without the deployment. If a mental disorder related to the deployment is identified, the diagnosis, name of the deployment, gender, partnership status, number of previous deployments, and age at the onset of the disorder are recorded. Diagnosis is completed according to ICD-10 following the procedures described in Section 2.2.

All individuals who were recorded in this register up to 31 December 2023 and who were diagnosed with a mental disorder related to the “Training Support Contingent Northern Iraq” deployment were extracted.

All data used in this study are anonymous. The soldiers recorded in the register are pseudonymized using an encryption system. This also prevents duplicate entries. In the rare cases where a mental disorder results from two different missions, only the first one is included in this database. Written consent was not required for this register study. This study was approved by the Ethics Committee of the University of the German Armed Forces in Munich (No.: 43 EK UniBw M 23-02). The publication of this study was approved by the Medical Command in Koblenz.

### 2.4. Merging the Two Data Registers

The key figures were extracted from both registers and combined. In this way, the cumulative incidence of deployment-related mental disorders among military personnel could be calculated. Since these are absolute figures, this was completed anonymously. Further methodological details are published elsewhere (Hüttermann et al., 2025).

### 2.5. Data Analysis

To calculate the cumulative incidence, the percentage of military personnel with deployment-related mental disorders was calculated from the total number of all deployed military personnel. In addition, descriptive statistics were compiled to illustrate the overall frequency of the main diagnoses and comorbidities, as well as an overview showing which main diagnosis is present in combination with which comorbidities. Subsequently, a chi-square test (χ^2^-test) was used to check whether the two most common deployment-related mental disorders differed with regard to the number of comorbid mental disorders.

In the next step, χ^2^-tests were used to examine whether there were differences in the frequency of mental disorders between ranks and between genders.

The latency period between the onset of the disorder and the initial diagnosis was calculated by simply subtracting the date of the initial diagnosis and the documented onset of the disorder at that initial diagnosis. To investigate whether there are gender differences in latency or whether there are differences in latency in PTSD compared to other disorders, *t*-tests were conducted. In order to determine the covariates number of previous deployments, age at onset of disorder, and partnership in case of significant results, an analysis of covariance (ANCOVA) was performed. Outliers were defined as Cook’s distance > 1. If outliers are identified and excluded, this is indicated accordingly. The residuals were tested for normal distribution using the Shapiro–Wilk test and the homogeneity of variances by Levene test.

All tests were carried out using IBM SPSS Statistics for Windows version 21.0 (Armonk, NY, USA).

## 3. Results

According to the central database on deployment-related mental disorders, by December 2023, *n* = 55 (3.4%) individuals had been recorded who developed a mental disorder as a result of this deployment. Of these, 83% lived in a partnership. With *n* = 28 (50.9%), PTSD was the most common main diagnosis followed by anxiety disorders (*n* = 18; 32.7%) and depressive disorders (*n* = 4; 7.2%). The number of all mental disorders, divided by main diagnoses and comorbidities, are presented in Table 2.

The next step was to investigate whether military personnel with one of the two most common mental disorders (PTSD and anxiety disorders) differ in the frequency of additional disorders. A significant difference was found with *χ*^2^ (1, *N* = 46) = 4.5, *p* = 0.033. Military personnel with PTSD as their primary diagnosis had significantly more comorbid mental disorders than their comrades with an anxiety disorder as their primary diagnosis. Table 3 shows which comorbid diagnoses are present for which main diagnoses.

To look for differences in rank, the cumulative incidences of mental disorders were compared by a 3 × 2 *χ*^2^-test. With *χ*^2^ (2, *N* = 1635) = 3.8, *p* = 0.152, there were no significant differences between ranks. With *χ*^2^ = 0.12, *p* = 0.727, there were no gender differences in the cumulative incidence (male 3.5%; female 2.8%) of mental disorders.

The latency period between the onset of disease and initial diagnosis was, on average, 1.0 years (standard deviation 1.1 years), and the quartiles (Q) were Q_25_ < 1 years, Q_50_ = 1 year, and Q_75_ = 2 years. In seven cases, the onset of the mental disorder was not recorded. With *t* (*N* = 48) = 0.58, *p* = 0.56, there were no significant differences in gender.

Comparing the latency period for PTSD as the primary diagnosis with other mental disorders, the period was longer in military personnel with PTSD (mean: 1.3 vs. 0.6 years): *t* (*N* = 48) = 2.3, *p* = 0.022. This result remained significant when controlling for the number of previous deployments, age at the onset of the disorder, and partnership, as shown in Table 4.

The homogeneity of the regression slopes was not violated with respect to the dependent variable, as the interaction terms were not statistically significant (*p* > 0.05). In all tests, *p* > 0.05 was obtained, meaning that the residuals can be considered normally distributed and the homogeneity of variances is fulfilled.

## 4. Discussion

We found a high cumulative incidence of mental disorders following the deployment of the Training Support Contingent to Northern Iraq (3.4%). Furthermore, the latency period between the onset of the mental disorder and initial diagnosis is significantly longer among service members with PTSD compared to their counterparts with other mental disorders.

We hypothesized that the cumulative incidence rate in Iraq would be lower than in Afghanistan. Although we did not test the hypothesis using statistical methods, the proportion of 3.4% of military personnel with deployment-related mental disorders is above our expectations. This high cumulative incidence rate is attributed to several significant threats. The presence of IS and other militant groups posed a direct threat, increasing the risk of suicide bombings, improvised explosive devices (IEDs), and small arms attacks on military facilities and military personnel. This threat was exacerbated by the proximity to the U.S. Embassy. The embassy was the target of regular attacks, mostly with rockets, from a distance. Due to the lower accuracy, the German camp also came under fire more frequently. This results in a higher proportion of deployment-related life-threatening incidents. Such incidents bear a six-to-seven times higher risk for the new onset of PTSD, depression, or an anxiety disorder (Wesemann et al., 2024).

Although the German service members were primarily deployed in training and support roles, they also encountered enemy forces in combat situations, particularly during operations close to the front lines. The volatile and rapidly changing nature of the conflict increased the risk of sudden attacks and ambushes and created a constant sense of danger. Dealing with the local population and Iraqi forces posed the risk of unintended civilian casualties, which could lead to moral dilemmas (Grimell, 2023; Grimell & Nilsson, 2020) and potential backlash (Adugna et al., 2024). These threats required a high degree of vigilance and adaptability from the military personnel during their mission. Over the long term, this also can increase the vulnerability to mental health problems (Hotopf et al., 2006; Reijnen et al., 2015; Wiborg et al., 2016).

The latency time from the new onset of the mental disorder until the first diagnosis with a median of one year is within our expectations. Nevertheless, the latency period was more than twice as long in military personnel with PTSD as in those with other mental disorders. A possible explanation could be the nature of the mission. While Afghanistan missions were seen as combat deployments with high media attention, Iraq was “only” a training mission with a low media presence. This could have been relevant for the perception of those affected by PTSD in reducing the likelihood of seeking professional help. This result highlights the importance of looking for this timeframe in other deployments. This knowledge is important for troop psychologists, military leaders, and the military personnel itself. It could lead to better screening and detection rates as well as to reduced stigma.

The area of external stigma is most likely to be present at the higher ranks of sergeants (Latza et al., 2018). The main attitudes that prevent those affected from seeking professional help are “not having a serious problem or being able to cope with it alone”, a “lack of effectiveness of psychotherapies”, “fear of stigmatization by others”, and “lack of logistical possibilities to seek help” (Siegel et al., 2018). Regular mental health assessments, as proposed by the “psychological fitness” approach, could alleviate this problem (Wesemann et al., 2018). Furthermore, involving family members can help reduce stigma (Wesemann et al., 2019). The use of the State-Trait Emergency Responder Questionnaire for Partners (STEP) is an approach to shortening the latency period (Hochfeld & Wesemann, 2025) that combines both approaches. Shortening the latency period is particularly important to avoid chronification, especially, in this case, because military personnel with PTSD often have comorbidities. This is likely to complicate treatment, may result in longer treatment times, and may also worsen the outcome.

Even so, female personnel had a somewhat lower cumulative incidence rate; there was no statistically significant difference. This result is supported by another study comparing gender differences in the one-year incidence rate of mental disorders after an Afghanistan deployment. There were also no significant differences, but there was an increase in hostility and problematic anger in female service members after deployment. This was expected as an early behavioral indicator for the later onset of PTSD (Wesemann et al., 2017). Nevertheless, even as a full assessment of the Iraq deployment, the number of participants is too small to drive concrete conclusions. This is truer as we do not have data on specific mental health symptoms.

More specific training tailored to the challenges of these missions could lead to better resilience (Doody et al., 2021) and better curricular training (Mortelmans et al., 2016). There are several programs with promising effects such as team-based skills training (Emaliyawati et al., 2025), team cohesion training (Tan et al., 2022), battlemind training (Castro et al., 2012), trauma risk management (Rona et al., 2017; Jones et al., 2019), or memory structuring interventions (Roberts et al., 2009). A currently highly regarded program is YaHaLOM. It was introduced in the Israeli Defence Forces to improve the functioning of decompensated military personnel (Svetlitzky et al., 2020). In a modified form, it was adopted by various armed forces, for example, by the German Armed Forces with BESSER or the US Armed Forces as iCOVER (Adler et al., 2020).

The cumulative incidence can be used in the future to identify risk factors (Tahernejad et al., 2024). This proactive approach could reduce the incidence of mental health symptoms (Maglione et al., 2022a; Nordstrand et al., 2024b). Nevertheless, there still seems to be room for improvement in primary prevention measures for PTSD, anxiety, or depression (Maglione et al., 2022b).

This is the first study examining the psychological consequences of deployed German military personnel in Iraq. Although the inclusion of just over 1000 individuals is neither large nor small, one of the strengths of this study is the full survey. Everyone deployed was included.

## 5. Limitations

This study only includes officially registered military personnel with a mental disorder related to their deployment in Iraq. The diagnostics correspond to the gold standard, so no false positives are to be expected. Despite the psychiatric assessment, it cannot be ruled out that some of the military personnel already suffered from a mental disorder before their deployment. However, the rate of individuals who remained undetected cannot be stated. Due to stigma or other barriers, the number of unreported cases can be relatively high. Due to the long latency period, the number seems to be higher. As 25% needed two and more years for being recorded, we still expect several individuals to be detected in the next few years.

Although this is a full survey, the sample size of affected military personnel is small. Therefore, the subgroup analyses, in particular, may contain bias and should be interpreted with caution.

Gender assignment was based on biological sex. However, the interpretations focus on gender. Therefore, there may have been deviations in the group assignment. However, the distortions are considered to be rather small. Given the high number of cases, this is unlikely to have had a significant impact on the results.

As this is a register study, no information about stigma, other barriers to care, or other relevant factors and covariates like unit cohesion, peer- and leader support, and support from family or friends concerning the results are available. Nevertheless, this information would be necessary to better understand and interpret the results.

The diagnoses were made by specialists using standard methods and thus correspond to the gold standard. However, diagnoses alone do not provide information about the extent of the damage, the impairment, the limitations in daily life, or the quality of life of those affected. As this study focuses exclusively on German personnel deployed to Iraq, the findings may not generalize to other operational contexts or national forces.

## 6. Conclusions

It is worth comparing the different missions in order to derive better preparedness and follow-up measures.

The type of deployment, the frequency and duration of deployments, and individual factors prior to deployment influence the results. By analyzing these variables, further risk factors can be identified. Screening can then be used to identify personnel at risk and provide them with special training.

The calculation of the cumulative incidence shows the long-term effects of mental health problems from the Iraq deployment. This has an impact on continued deployment, the reintegration into civilian life, their relationships, and their general quality of life. Documenting the frequency of these disorders is intended to help highlight the need for ongoing support. These findings are relevant for the development of long-term strategies that go beyond the immediate interventions after deployment. This increases the chance that the soldiers will also receive the necessary resources in the long term. The Bundeswehr’s current approach is the concept of “psychological fitness” (Wesemann et al., 2018), which, in addition to regular screenings, also enables comprehensive, results-based health promotion measures. This is comparable to the US’s “Combat Stress Control (COSC)” concept (Bruscher, 2011; Maglione et al., 2022b), the UK’s “Trauma Risk Management (TRiM; Greenberg et al., 2008)”, or the Canadian Armed Forces’ “Road to Mental Readiness (R2MR)” (Fikretoglu et al., 2019).

Understanding cumulative incidence contributes to the broader field of military psychology and public health. By providing empirical data on mental health outcomes associated with specific deployments, evidence-based policies can be developed. This can be used as a basis for setting foreign assignment allowances. It can also influence decisions about funding, program development, and resource allocation.

In addition, assessing cumulative incidence promotes a culture of transparency and accountability within the military. By openly acknowledging mental health issues and disorders as an occupational hazard, military leadership can foster a more supportive environment. This change helps reduce stigma and encourage help-seeking behavior among employees. When soldiers and supervisors see that their organization is committed to treating mental health issues based on empirical data, it can lead to vicarious learning.

Future research in this area could obtain representative samples from deployments during the course of their deployment. This could include screening military personnel for mental disorders before and after deployments using clinical interviews. Combining this research strategy with the one presented here would make it possible to capture the utilization of the support of those affected. This would also enable the assessment of changes and thus the effects of destigmatization processes over time. The different developments of mental health symptoms across different deployment areas should also continue to be the focus of research. More individualized diagnostics enable adjustments and thus more targeted support services before and after deployment, as well as measures to destigmatize.

## Figures and Tables

**Table 1:** Demographics of military personnel deployed to Iraq between 2015 and 2018.

Deployed Personnel	Male	Female
Enlisted soldiers	132	0
Sergeants	855	56
Officers	540	52

**Table 2:** Number of mental disorders and comorbidities resulting from the deployment of the Training Support Contingent Northern Iraq.

Mental Disorder	N First (Main) Diagnosis	N Second (Comorbid) Diagnosis	N Third (Comorbid) Diagnosis
Depressive disorders	4	6	5
Anxiety disorders	18	12	3
PTSD	28	2	0
Alcohol dependence	0	2	0
Else	5	3	1

N = number of military personal with a main or comorbid diagnosis.

**Table 3:** Crosstabulation illustrating which primary diagnosis has which comorbid diagnosis for military personnel deployed to the Training Support Contingent Northern Iraq mission.

Main Diagnosis		Comorbid	Diagnosis		
	Depression	Anxiety	PTSD	Alcohol	Else
Depression	0	0	2	0	0
Anxiety	1	1	0	0	1
PTSD	5	10	0	3	1
Else	0	1	0	0	0

**Table 4:** ANCOVA to test the influence of PTSD on the latency period between the new onset of the mental disorder and first diagnosis, including number of previous deployments, age at onset of the disorder, and partnership as covariates.

Predictors	Df	F	Sig.	Ƞ^2^
Number of previous deployments	1	1.12	0.296	0.027
Age at onset of the disorder	1	0.07	0.801	0.002
Partnership	1	0.12	0.731	0.003
PTSD	1	4.14	0.048	0.092

Note. Df: degrees of freedom; F: F-value; Sig.: significance; R^2^ = 0.12; ƞ^2^: partial eta square.

## Data Availability

Data requests can be directed to the corresponding author. They are then checked on a case-by-case basis and require the approval of the Federal Ministry of Defence.
